# A Genome-Wide Survey of Date Palm Cultivars Supports Two Major Subpopulations in *Phoenix dactylifera*

**DOI:** 10.1534/g3.115.018341

**Published:** 2015-05-08

**Authors:** Lisa S. Mathew, Michael A. Seidel, Binu George, Sweety Mathew, Manuel Spannagl, Georg Haberer, Maria F. Torres, Eman K. Al-Dous, Eman K. Al-Azwani, Ilhem Diboun, Robert R. Krueger, Klaus F. X. Mayer, Yasmin Ali Mohamoud, Karsten Suhre, Joel A. Malek

**Affiliations:** * Genomics Laboratory, Weill Cornell Medical College in Qatar, Qatar Foundation, 24144 Doha, Qatar; ‡ Department of Genetic Medicine, Weill Cornell Medical College in Qatar, Qatar Foundation, 24144 Doha, Qatar; § Weill Cornell Medical College in Qatar, Qatar Foundation, 24144 Doha, Qatar; † MIPS/IBIS, Helmholtz Zentrum München, 85764 Neuherberg, Germany; ** USDA-ARS National Clonal Germplasm Repository for Citrus and Dates, University of California, Riverside, California 92507

**Keywords:** date palm, domestication, genotyping-by-sequencing, population genetics, plant sex chromosomes

## Abstract

The date palm (*Phoenix dactylifera* L.) is one of the oldest cultivated trees and is intimately tied to the history of human civilization. There are hundreds of commercial cultivars with distinct fruit shapes, colors, and sizes growing mainly in arid lands from the west of North Africa to India. The origin of date palm domestication is still uncertain, and few studies have attempted to document genetic diversity across multiple regions. We conducted genotyping-by-sequencing on 70 female cultivar samples from across the date palm–growing regions, including four *Phoenix* species as the outgroup. Here, for the first time, we generate genome-wide genotyping data for 13,000–65,000 SNPs in a diverse set of date palm fruit and leaf samples. Our analysis provides the first genome-wide evidence confirming recent findings that the date palm cultivars segregate into two main regions of shared genetic background from North Africa and the Arabian Gulf. We identify genomic regions with high densities of geographically segregating SNPs and also observe higher levels of allele fixation on the recently described X-chromosome than on the autosomes. Our results fit a model with two centers of earliest cultivation including date palms autochthonous to North Africa. These results adjust our understanding of human agriculture history and will provide the foundation for more directed functional studies and a better understanding of genetic diversity in date palm.

As one of the oldest cultivated fruit trees, the date palm is intimately tied to the history of human migration. Based on the archeological record, its domestication, or earliest cultivation, is frequently believed to have occurred in the fertile crescent (Zohary and Spiegel-Roy 1975). Archaeological evidence points to its regular cultivation by the 4^th^ to early 3^rd^ millennium B.C. (Tengberg 2012). It is a monocotyledon tree from the family Arecaceae (Palms) that also includes the commercially important Oil and Coconut Palms. The Phoenix genus contains 14 species (Barrow 1998) that are endemic to various regions stretching from islands off West Africa to East Asia. Hybridization among the species does occur (Balthazard 2013) and dioecy, the presence of separate male and female trees, offers the opportunity for high genetic diversity once hybridization has introduced new genetic material.

Numerous hypotheses for the origin of date palm and the location of its domestication have been presented with little conclusive results (Gros-Balthazard *et al.* 2013). These include a proposed derivation from an original wild species or a derivation from *P. sylvestris*, among others. Most recently, genetic analysis of *Phoenix* chloroplasts suggested that *P. dactylifera* is genetically distinct enough to likely not have been domesticated from other Phoenix species (Pintaud *et al.* 2010). This observation is also supported by morphometric analysis of date palm seeds that clearly differentiated *P. dactylifera* from multiple other *Phoenix* species (Terral *et al.* 2012; Rivera *et al.* 2014). The geographical origin of the date palm is equally inconclusive, with the earliest archeological evidence of cultivation coming from sites around the Arabian Gulf (Tengberg 2012). The Indus Valley and Fertile Crescent in general are also seen as possible sites (Tengberg 2012). Additionally some have suggested East Africa/South Arabia as a possible site of domestication (Larson *et al.* 2014). The existence of multiple domestication centers and multiple domestication events has also been considered (Terral *et al.* 2012; Pintaud *et al.* 2013). Confounding the research is the fact that for centuries date palm, despite its ability to outcross, has been cultivated using clonal propagation from off-shoots or tissue culture. The purpose for this is to maintain elite varieties that produce uniform fruit quality. Further confounding research on domestication is the movement of elite cultivars that has occurred across the region of cultivation.

The questions about date palm domestication remain of high-interest because it will provide information in the search for genes under selective pressure from human intervention such as farming. To address this, Gros-Balthazard and colleagues called for genetic surveys of date palm samples taken from across the world-wide zone of cultivation (Gros-Balthazard *et al.* 2013). They noted that surveys to date have been of local genetic variation limited to specific regions with a few exceptions (Arabnezhad *et al.* 2012; Zehdi *et al.* 2012). Rhouma-Chatti Soumaya (2014) sampled date palm cultivars in Tunisia and included some that originated from the Arabian Gulf. Utilizing AFLP and RAMPO markers, they did not detect a strong signal for genetic separation of their samples based on geographical origin. A genetic survey was conducted of a large set of date palm cultivars (Chaluvadi *et al.* 2014); however, most of the varieties were from the Arabian Gulf region. In contrast, recent surveys that have included date palms from at least two different regions have shown data supporting two distinct genetic backgrounds from North Africa (West) and the Arabian Gulf (East) (Arabnezhad *et al.* 2012; Zehdi *et al.* 2012). Furthermore, studies of the XY chromosome have identified similar separation with two distinct Y alleles segregating between Eastern and Western cultivars (Cherif *et al.* 2013). Some studies using methods such as metabolomics or morphometry have not shown a clear separation between the two geographical groups (Khierallah *et al.* 2011; Terral *et al.* 2012; Farag *et al.* 2014). To our knowledge, all genetic analyses of date palm to date have utilized Simple Sequence Repeat (SSR) markers or similar methods with low marker density. Genome-wide single nucleotide polymorphism (SNP) analysis has not been conducted to assess date palm genetic diversity. Until recently, the date palm lagged behind other important crops in genetic resources, despite its importance. Recent projects have produced a genome sequence (Al-Dous *et al.* 2011; Al-Mssallem *et al.* 2013), a first genetic map (Mathew *et al.* 2014), and the identification of the putative sex chromosome of the date palm (Mathew *et al.* 2014). These resources provide the necessary foundation for further genetic studies.

To address the paucity of geographically diverse genomic analyses, we collected samples from various date palm cultivating countries as an initial survey of genetic diversity in *P. dactylifera*. The number of date palm cultivars is estimated to be in the thousands (Johnson 2011); however, for practical purposes, we collected samples that were of mainly commercially important cultivars. We chose to collect either leaf or, for the first time in large scale, fruit samples. The date fruit mesocarp derives from the female ovary and does not integrate genetic material from the male pollinator (see *Results*). Collecting fruit samples ensures that the collection is based on the commercially important phenotype (fruit type) and allowed documentation of phenotypic information such as fruit size, color, and weight for future analysis. Because fruit is one of the clearest phenotypes in date palm, we chose to exclude the use of males in this study because proving their geographical origin is difficult. Due to the fact that various countries use similar names for different fruit or different fruit may have the same name (Popenoe 1913; Terral *et al.* 2012), we opted to include some samples with similar names. Our goal was to identify population structure in the collected samples using the first application of genome-wide SNP analysis to date palm genetic diversity. These results would help guide future research to identify representative cultivars for certain regions and genes critical to the cultivation of date palm.

## Materials and Methods

### Date fruit sample collection and preparation

Date fruit were collected from commercial outlets across the region. In addition to eight date palm samples from leaves already sequenced (Al-Dous *et al.* 2011), we collected 62 fruit samples originating from 10 different countries (Supporting Information, Table S1). Although packaging of the dates reveals the country of production, the genetic origin of these date palm fruit cannot be confidently pinpointed to a country. For example, Medjoul dates were collected from Saudi Arabia and from California, although the genetic origin is likely Morocco/Algeria (Sedra 2011). Given that most date palm cultivars have Arabic names, there are multiple transliterations for many of the cultivars collected. Name, location of cultivation, origin, and other information were collected for each cultivar (Table S1). Multiple dates from each cultivar were stored for future phenotypic analysis. Date fruit samples were washed multiple times in distilled water and cut open, the seeds were removed, and 200 mg of mesocarp was crushed with the TissueLyser II (Qiagen). DNA was extracted with the DNeasy 96 Plant Kit (Qiagen) according to the manufacturer’s protocol. As an internal control for the process, DNA extraction and genotype analysis were performed twice for four samples. DNA was quantified and normalized using a Nanodrop (ThermoScientific). For four fruit samples, two DNA extractions were repeated from the same fruit sample and passed through the entire genotyping and analysis process to provide an internal quality control.

### Genotyping-by-sequencing

Genotyping-by-sequencing (GBS) libraries were constructed as described (Mathew *et al.* 2014) including the use of size selection to ensure that SNPs were sequenced to a higher level of coverage for accurate heterozygous SNP calling. Specifically, after pooling of 24 barcoded sample libraries, the pools were size-selected on a 1.5% agarose gel from 350–550 bp using the automated Pippin Prep system (Sage Science, USA). Size-selected samples were then amplified by PCR for 16 cycles and cleaned with a 1.2X Agencourt AMPure XP (Beckman Coulter, USA). Pools containing 24 libraries were sequenced on separate lanes of the HiSeq2500 (Illumina, USA) according to the manufacturer’s paired-end sequencing protocol. Approximately 360 M paired-end reads were collected per lane. Libraries were "spiked" with 20% of a balanced nucleotide library to improve base-calling within the barcode region of the sequence reads.

DNA sequences were separated for each barcoded library and matched to the Date Palm V3 genome (http://qatar-weill.cornell.edu/research/datepalmGenome/download.html) using BOWTIE2 (Langmead *et al.* 2009). SNPs were then called with SAMTOOLS (Li *et al.* 2009) to generate a VCF file. Insertions/Deletions were not included and sites were only allowed to have two possible alleles. We applied stringent filtering on raw SNP calls to ensure high-quality genotypes (minimum 10× sequence coverage in at least 80% of individuals). Genotypes with a PL score, as determined by SAMTOOLS, of less than 35 for any secondary call were considered missing and only alternative alleles observed at least four times for a site were included as polymorphic.

### Population structure analysis

STRUCTURE (Pritchard *et al.* 2000) was run with standard settings. All runs included a 5000 burn-in period and 25,000 MCMC Repetitions. Populations reached stable *Fst* levels well within the 5000 burn-in periods. We ran STRUCTURE twice, first to identify population structure in the date palm cultivars only and second, including the *Phoenix* species, to identify the date palm cultivar relationship to *Phoenix* species in general.

In the case of population structure among the date palm cultivars, we ran STRUCTURE for K (number of populations) set from 2 to 7, with 4 replicates for each K (Table S2). We then re-ran STRUCTURE 12 times with K set to 2. STRUCTURE HARVESTER (Earl and von Holdt 2011) was used to identify the optimal number of clusters. CLUMPP (Jakobsson and Rosenberg 2007) was used to summarize the results of multiple STRUCTURE runs. For inclusion of Phoenix species, we ran STRUCTURE 10 times with a K set to 3. Principal components analysis was conducted in Partek Genomics Suite (PARTEK, USA) by assigning homozygous reference alleles as 1, heterozygous as 2, and homozygous alternative alleles as 3.

### Phylogenetic analysis

Multiple alignments were constructed from genotype data by concatenating all SNPs (see File S1). The tree topology was determined based on homozygous SNPs by applying FastTree (Price *et al.* 2009) to 100 bootstrapped samples. The consensus tree was constructed using the consense tool from the PHYLIP package (Felsenstein 1989). Distances were computed with the dnapars tool providing the topology derived previously as input. The tree was plotted using the iTOL (Letunic and Bork 2011).

### Private allele analysis

Private alleles are defined as alleles that occur exclusively in one of the two populations. Heterozygous positions were handled by considering both alleles independently for all positions. No additional filters regarding call rate or private allele frequency were applied. Distribution of private alleles on linkage groups was calculated by constructing artificial chromosomes following the contig order in the genetic map.

## Results

### Genotyping-by-sequencing of date palm fruit and *Phoenix* outgroup samples

Using genotyping-by-sequencing we genotyped 74 *Phoenix* samples representing 70 date palm samples, of which 62 were distinct fruit samplings. In addition, we included four other Phoenix samples from two *Phoenix hanceana* (*loureiroi*) and two *Phoenix sylvestris* trees in the USDA collection in California.

We used various cutoffs for allowed numbers of missing genotypes depending on the analysis. For phylogenetic tree analysis, we allowed a maximum of 10 missing genotypes at a given SNP position and included only *P. hanceana* as the most distant outgroup, resulting in a set of 26,846 SNPs passing cutoffs (see File S1). For date palm population STRUCTURE analysis, we included both *P. hanceana* and *P. sylvestris* and allowed a maximum of eight missing genotypes for a given SNP position. This resulted in 12,995 SNPs passing cutoffs (see File S2). For allelic differences between populations, we included only the 70 date palm cultivars and allowed a maximum of 15 missing genotypes at a given SNP position. This resulted in a total of 67,496 SNPs passing cutoffs (see File S3).

Analysis of the duplicated samples and a comparison of both Medjoul and Khalas date fruit to their respective whole genomes (Al-Dous *et al.* 2011), derived from leaves, revealed a predicted genotyping accuracy of 99.4% (assuming errors are equally likely in both samples). This suggests that genotyping accuracy is high and that DNA extracted from the fruit samples indeed matches DNA from the leaves with minimal contamination in general.

### Phylogenetic relationship of date palm cultivars

**Figure 1:**
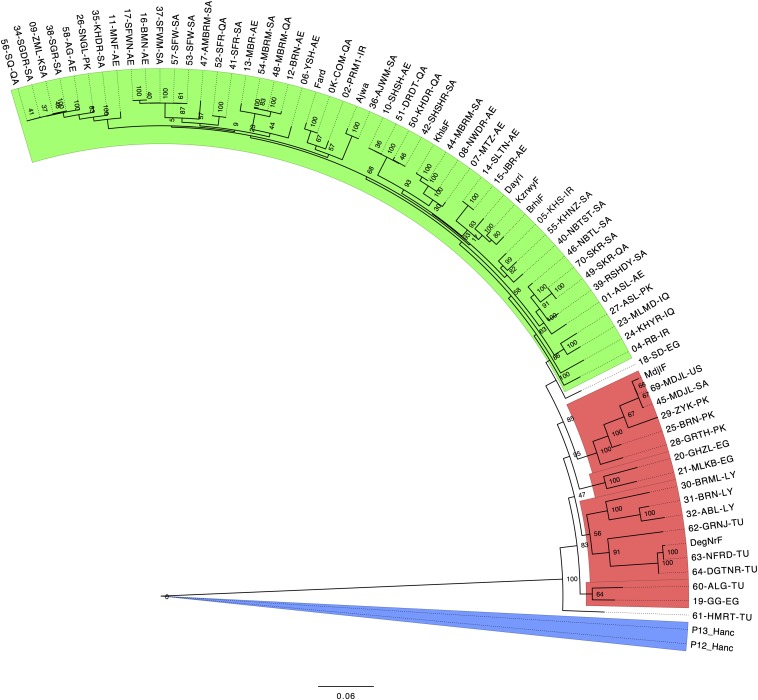
Phylogenetic tree of date palm cultivars using *P. hanceana* as outgroup. Genotyping information was used to construct a consensus tree of date palm cultivars. Eastern cultivars (Arabian Gulf) are highlighted in green, Western cultivars (North Africa) are in red, outgroup (*P. hanceana*) is in blue, and the boundaries are left white. Branch labels are cultivar IDs (Table S1). Node labels are bootstrap values from 100 runs. There is well-supported separation for the division of Western and Eastern cultivars. However, some divisions within date cultivars from geographically related regions have low bootstrap support.

We constructed a phylogenetic tree based on multiple alignments of genotyped SNPs, including two *P. hanceana* samples as outgroup. Bootstrapping revealed that major branches were well-supported especially for the division of the date palm cultivars into Western and Eastern cultivars (Figure 1). Some branches within geographic regions had lower bootstrap support. Multiple samples from the same cultivar, collected in different locations, regularly clustered near each other. For example, Medjoul collected in California from fruit and leaves at different times and Medjoul collected from Saudi Arabia clustered together on the tree. Other examples include Mabroom and Safawi cultivars. Conversely, some cultivars with the same name did not cluster including the Mabroom from Saudi Arabia that did not cluster with Mabroom samples collected from Qatar and the United Arab Emirates, and a second Saudi Arabia Mabroom sample. This may be due to the use of seed-grown cultivars or misnaming. An interesting observation is that some Pakistani cultivars are located in the Western date palm cultivar group. Specifically, the Zayaki, Barani, and Gorakh varieties appear to be derivatives of Medjoul. Genetic analysis of relatedness using VCFTOOLS (Danecek *et al.* 2011) supported this conclusion (results not shown) and likely reflects the creation of Pakistani cultivars by local pollination of the elite Medjoul imported from Morocco/Algeria. This use of seed propagated date palms is common in Pakistan and results in cultivars of mixed genetic background (Milne 1918). Altogether, this reinforces the observation that sometimes cultivars with the same name and similar appearance may differ genetically (Terral *et al.* 2012).

The closest date palm cultivars to the *P. hanceana* outgroup are from the Western (North African) group (Figure 1). Increased levels of introgression in the Western cultivars are unlikely because *P. hanceana* is indigenous to southern Asia (Barrow 1998) and therefore geographically separated from the Western cultivars by the Eastern date palm cultivar region.

### Subpopulation structure in date palm

**Figure 2:**
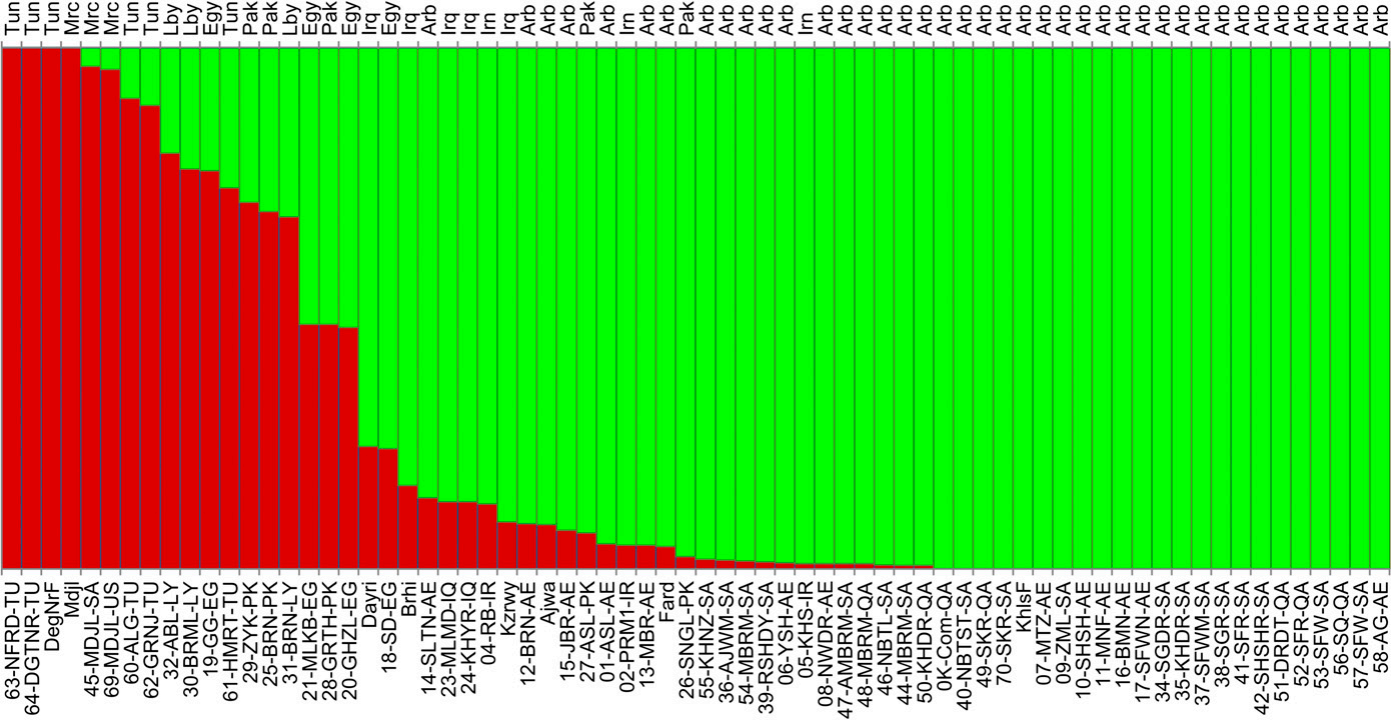
Date palm cultivar genetic structure. STRUCTURE was run assuming a population size of 2 and each cultivar was assigned a population proportion (see *Materials and Methods*). Proportions were plotted with the sample ID below and assumed country of genetic origin above (for country names see Table S1). United Arab Emirates, Oman, Saudi Arabia, and Qatar were collapsed into "Arb" for "Arabia." Samples were organized by country and by sub-population proportion. Mixed samples are both in the middle of the date palm growing region (Egypt, Libya) but also originate from Pakistan. Additional analysis suggests these samples from Pakistan are likely from a recent breeding with the elite Medjoul (Western) variety.

For the analysis of population structure among the date palm cultivars, we used the STRUCTURE program (Pritchard *et al.* 2000) to identify numbers of subpopulations and their genetic constitution. Analysis of the results using STRUCTURE HARVESTER (Earl and von Holdt 2011) showed the strongest support for two populations, which is consistent with the phylogenetic tree analysis. The strongest division among date palms is between Eastern (Arabian Gulf) and Western (North Africa) cultivars (Figure 2). A group of admixed cultivars is in the center and includes cultivars from Egypt that are likely admixed simply due to their location between the two major regions (Pintaud *et al.* 2013), in addition to cultivars from Pakistan that appear to be recent breeds of Medjoul (discussed above).

To better understand the relationship of the date palm subpopulations to their ancestor, we ran STRUCTURE while including four samples of other *Phoenix* species: two independent samples of *P. hanceana* (*loureiroi*) and two independent samples of *P. sylvestris*. *P. hanceana* is believed to be among the most distant *Phoenix* species from *P. dactylifera*, whereas *P. sylvestris* is believed to be among the closest species (Pintaud *et al.* 2010). We ran STRUCTURE under the assumption of three populations (Hubisz *et al.* 2009) by providing the program with the expectation that the *Phoenix* species would form the third subpopulation and reveal their influence on the eastern and western cultivar groups. CLUMPP summary of the 10 runs gave an H′ of 0.93, indicating good concordance among the 10 independent runs.

**Figure 3:**
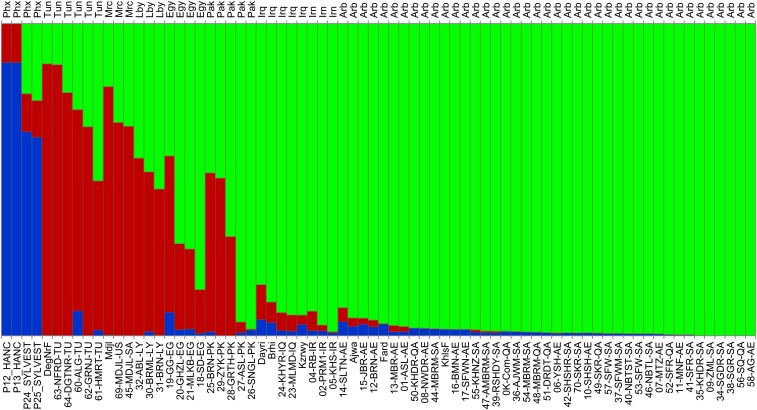
Date palm cultivar genetic structure in the context of other Phoenix species. See Figure 2 for details. STRUCTURE was run assuming a population size of 3 using the sampling locations as in Figure 2 with locations set as East, West, Mix, and Phoenix species. Phoenix species were plotted on the left. Western cultivars show a very specific shared (red) set of alleles, whereas the Eastern cultivars show a sharing of the blue alleles. Western varieties show a higher shared proportion with the Phoenix species *vs.* the Eastern cultivars. Eastern cultivars to the far right of the plot essentially shared no ancient alleles of *P. hanceana*.

The bulk of the genetic structure in the *Phoenix* outgroup species derives from alleles common among them yet differing from the *P. dactylifera* samples (Figure 3). *P. hanceana*, one of the Phoenix species most distant from date palm, shared *P. dacytlifera* alleles mainly with the Western date palms (Figure 3, red bar), whereas *P. sylvestris* showed higher overall allele sharing with *P. dactylifera*. These shared alleles were distributed between the North African and the Arabian Gulf groups. Although most date palm samples from North Africa and the Arabian Gulf contained some proportion of the alleles common among the *Phoenix* outgroup, five date fruit samples from the Arabian Gulf region showed no sharing of alleles common among the ancestral species (Figure 3, fully green).

The North African cultivars, in general, showed higher levels of allele sharing with the distant Phoenix species (Figure 3). Western North Africa (Morocco, Tunisia, Libya) had the highest levels of shared alleles, whereas samples from the Arabian Peninsula (Saudi Arabia, Qatar, United Arab Emirates) had the lowest levels. As previously noted, the natural habitat of *P. hanceana* is among the furthest east (Southern Asia) (Barrow 1998) of the *Phoenix* species, and introgression would most likely have occurred with the Eastern Date Palm varieties. This may suggest that the observed allele sharing with North African cultivars is the increased retention of the ancestral allele in the western date palm.

**Figure 4:**
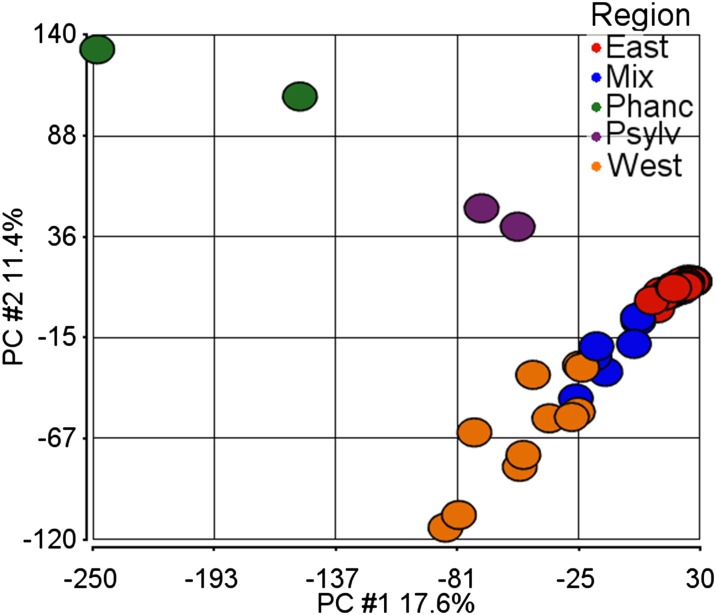
PCA analysis of date palm cultivars in the context of other Phoenix species. Cultivars are colored by their region according to STRUCTURE analysis. "East/West" are defined by having at least 75% purity, as determined by STRUCTURE, for their respective regions. "Mix" samples have less than 75% purity for any given region. "Phanc" is *P. hanceana* and "Psylv" is *P. sylvestris*. The first component accounts for 17.6% of variance and separates the western date palm cultivars closer to the outgroups *P. hanceana* and *P. sylvestris*. The second component accounts for 11.4% of the variance and clusters eastern date palm cultivars closer to the out species.

For concordance, we ran Principal Components Analysis (PCA) on the same data set including the 70 date palm cultivars and four samples from outgroup *Phoenix* species (Figure 4). The first and second components were responsible for 17.6% and 11.4% of the variance, respectively. The PCA yields results similar to those of the Phylogenetic and STRUCTURE analyses. The first component separates the western date palms with the *Phoenix* species, whereas the second component separates the eastern cultivars with the other species (Figure 4).

We calculated *Fst* (Weir and Cockerham 1984) on the 67,496 SNP data set. Based on the STRUCTURE analysis (discussed above), date palm cultivars were assigned to the Western or Eastern cluster if they showed at least 75% purity for that cluster. This resulted in 11 samples included in the Western clusters and 52 samples included in the Eastern clusters. Seven samples were declared "mixed" and assigned to a Central region (Table S1). When samples from the Western and Central (Mixed) regions were considered, the mean and weighted *F_st_* were 0.159 and 0.214, respectively. When Western and Eastern populations alone were compared, the mean and weighted *F_st_* were 0.208 and 0.267, respectively. This suggests that the more distant Western and Eastern populations show significant separation with low levels of breeding between them, as expected based on their geographical separation. These results agree favorably with those found by Zehdi *et al.* (2012) when comparing various North African date palm cultivars to a small sampling of Arabian Gulf cultivars.

### Allelic differences in date palm subpopulations

**Figure 5:**
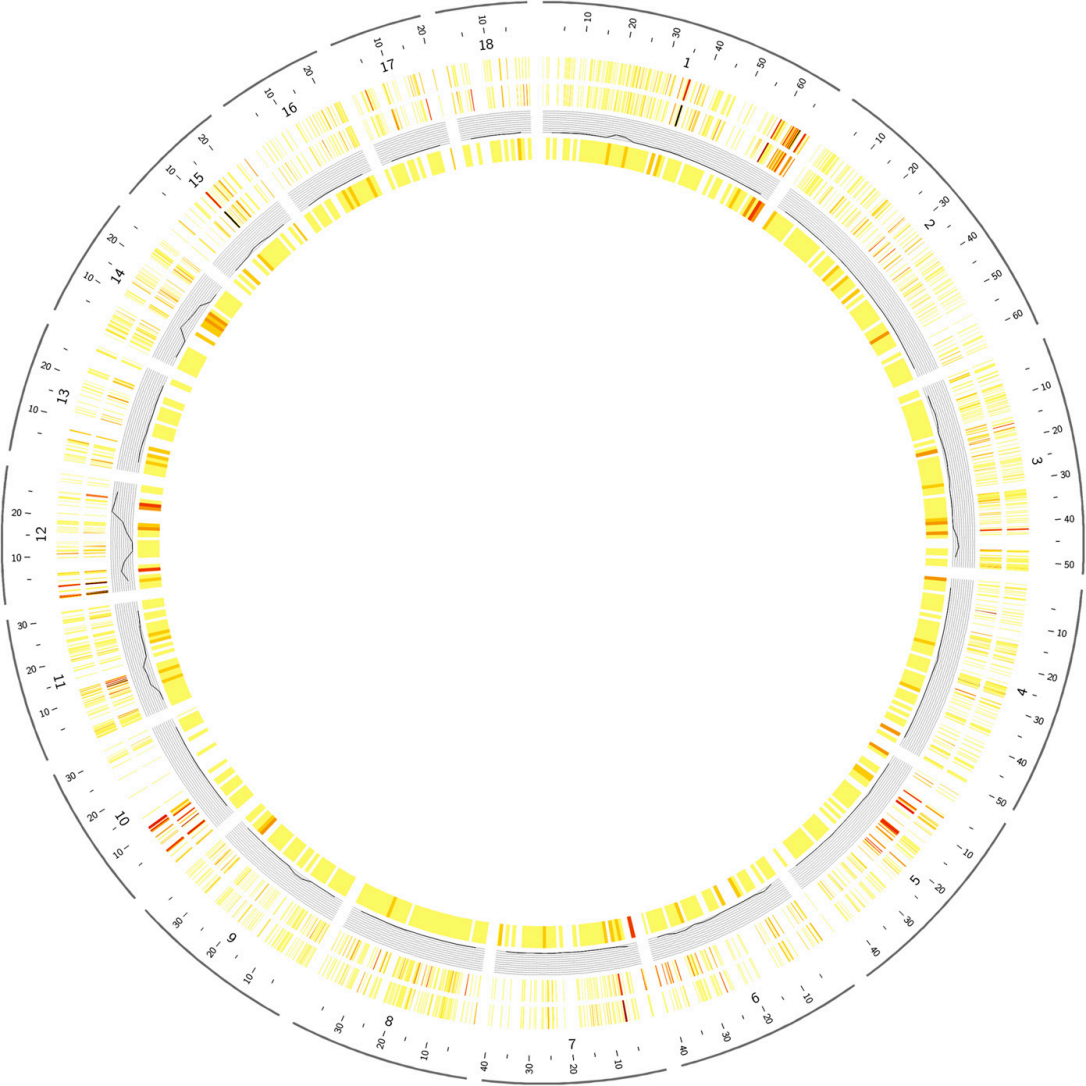
Genome distribution of various SNPs differing between Western and Eastern date palm populations. The distribution of SNP differences were plotted across the genetic map of date palm. Coloring of the figure is based on the number of private alleles found in the respective contig (yellow lowest, brown highest). Starting from the outer track: (1) the density of Western date palm population private alleles; (2) density of Eastern population private alleles (tracks 1 and 2 show some hotspots with increased counts of population private SNPs, especially on LG1, LG10, and LG15); (3) a line plot of SNPs fixed between Western or Eastern populations; and (4) a density plot of SNPs at significantly different allele frequencies between Western and Eastern date palm populations.

We screened for population-specific alleles using 67,496 SNPs to better understand the effects of population division and regional selective pressures on date palm genetics. Multiple forms of allele differences between the subpopulations including alleles that were private to one subpopulation, alleles that were overrepresented in either subpopulation, and alleles that appeared to have reached fixation differently between the subpopulations were analyzed (Figure 5).

Alleles occurring exclusively in one subpopulation between the combined Western plus Mixed group *vs.* the Eastern cultivars were investigated. As discussed above, date palm cultivars were assigned to the Western or Eastern cluster if they showed at least 75% purity for the STRUCTURE results, with the remainder assigned to a mixed category (Table S1). The mixed group included cultivars from Egypt, Libya, and Pakistan that we combined with North Africa in this analysis. Of 67,496 variant positions, 23,988 (36%) qualify as nonreference private alleles, with 15,302 originating from the Western or Mixed population and 8686 originating from the Eastern population. This imbalance between the two is expected given that the reference sequence is derived from the Eastern cultivar "Khalas." The majority of private alleles that segregate the populations have not reached fixation.

Although private SNPs are only present in one subpopulation, we also identified alleles that were overrepresented in one subpopulation. Alleles at each position were counted and a simple Fishers exact test was applied to detect allele frequency differences between the two populations. We applied the Bonferroni correction to *P* values, and a corrected *P* < 0.01 was considered significant. Using this cutoff we obtained 3219 SNPs (4.8%) considered to be significantly segregating between the Western and Eastern date palm populations. This large number of alleles at different frequencies between the two populations reinforces the observation of significant Western to Eastern population structure observed in *F_st_*, PCA, and STRUCTURE analysis.

To detect whether some Linkage Groups (LGs) of the genome displayed higher levels of population-specific allele frequencies, we plotted the number of SNPs with significantly deviated frequencies between Western and Eastern clusters *vs.* the total number of SNPs for each LG (Figure S1). This revealed that LG 12 has approximately three-fold more SNPs with different allele frequencies between the Western and Eastern Date Palm cultivars than expected by the genome average. We previously determined LG 12 to harbor the putative sex determination region in a nonrecombining region spanning a large section of this linkage group (Mathew *et al.* 2014). The large numbers of SNPs with population-specific allele frequencies are not just localized to the sex determination region because we observed their elevated numbers across the whole linkage group. To study the influence of the elevated number of region-specific SNPs on population structure in date palm, we ran the program STRUCTURE on a data set excluding all 574 SNPs from LG 12. Using parameters described previously, the strongest support was found for three subpopulations (Figure S2). The major North Africa/Arabian Gulf divide still represented the major subpopulation fractions; however, the Arabian Gulf showed a second minor population, with the highest fraction individuals being from Iraq. This may suggest a second genetic influence on the Arabian Gulf date palms.

Whereas LG 12 showed elevated fixation, LG 8 showed approximately half the number of expected significant SNPs given the genome average. A possible reason for this may be selective sweeps for the preservation of domestication alleles on this chromosome that are equally critical to both geographical regions of cultivation. Further work will be necessary to better understand the reason and possible functional implications of decreased or increased allele fixation on a specific linkage group.

Scaffolds from the genome that showed elevated levels of private SNPs between Western and Eastern populations were further investigated. Using previously sequenced genomes (Al-Dous *et al.* 2011), we analyzed SNPs that segregated completely with Eastern (Khalas) or Western (Deglet Nour and Medjoul) cultivars. Although the number of whole-genome sequences is too few for comparison, large stretches of deviation between them may be of functional importance. We checked these polymorphic positions against the Oil Palm genome sequence to attempt identification of the ancestral allele. Some scaffolds did indeed show elevated levels of SNPs that both segregated with region and showed a preponderance of one region’s SNPs to deviate from the Oil Palm genome sequence. As an example, we plotted the scaffold PDK_30s1127551 (Figure S3), which contains two predicted genes and that showed elevated numbers of SNPs segregating between Western and Eastern cultivars. Of these 32 SNPs showing geographical segregation, 25 SNPs (78%) showed the Eastern cultivars had deviated from the ancestral allele in Oil Palm while the Western varieties had maintained the Oil Palm allele. Two SNPs (6%) showed the Western cultivars had deviated from the ancestral allele. One SNP (3%) showed both Western and Eastern alleles had deviated from the oil palm sequence. This last category likely represents some of the most ancient *Phoenix* or date palm–specific polymorphism and will be of interest for future studies. For the remaining four SNPs (13%), the surrounding sequence could not be unambiguously aligned to the oil palm genome sequence and were not scored. Regions showing elevated segregation of SNPs by geographical region may offer a starting point for understanding differences in cultivation and environmental pressures between North Africa and Arabian Gulf date palms.

## Discussion

The date palm remains an important fruit tree to a large section of arid land stretching from western North Africa to western regions of India. The earliest archaeological evidence for date palm cultivation is found in the area surrounding the Arabian Gulf (Tengberg 2012), and this region is widely believed to be the origin of date palm domestication. Whether the date palm has been domesticated has been a matter for debate because no wild progenitor has conclusively been identified (Gros-Balthazard *et al.* 2013). Recent efforts at documenting date palm seed morphology in suspected wild date palms would suggest that date palm cultivation has led to significant changes in traits such as seed shape (Terral *et al.* 2012). Under these observations the date palm has indeed been domesticated, although the effects may not be as dramatic when compared to other species (Purugganan and Fuller 2009). Despite the archaeological evidence for an early center of cultivation around the Arabian Gulf, some recent evidence has shown two distinct genetic subpopulations of date palm in the Arabian Gulf and North Africa (Arabnezhad *et al.* 2012; Zehdi *et al.* 2012). Some conflicting evidence has shown little genetic structure between the regions (Sedra *et al.* 1998; Khierallah *et al.* 2011).

Our work is the first to analyze genome-wide SNP genotype data in date palm cultivars and strongly supports the model of at least two distinct independent regions of earliest date palm cultivation centered in North Africa and the Arabian Gulf. These findings support a recent analysis that has suggested a similar model. Interestingly the samples collected by Zehdi *et al.* (2012) are largely of the North African type, with a few samples from the Arabian Gulf. In our case, the major proportion of samples is from the Arabian Gulf. Despite these differences, similar observations of two distinct groups with different genetic origins were reported. Phylogenetic tree analysis, STRUCTURE analysis, and Principal Components Analysis show that some North African date palm cultivars are among the closest to the *Phoenix* outgroup. This is unlikely due to a modern introgression as the *P. hanceana* habitat is separated from the North African cultivars by the Arabian Gulf cultivar region. Samples from Tunisia and Libya were at the base of the date palm Phylogenetic tree. Deeper sampling from this region will be required to identify whether this region in general holds date palms with higher diversity and more ancient alleles.

We have shown that approximately 36% of SNP locations contain alleles private to either date palm subpopulation. This highlights the strong genetic separation of the subpopulations. Whether the genetic differences in subpopulations of date palms in North Africa and the Arabian Gulf are due to differentiation during extended isolation or different domestication events remains a question. It is also possible that a single domestication occurred with various hybridizations to endemic species for each region, as may have happened in the domestication of rice (Londo *et al.* 2006; Huang *et al.* 2012). The maintenance of various ancestral haplotypes that we observed between the geographical subpopulations may indicate independent domestication events or differing selective pressure based on geography, as was observed in rice (Tang *et al.* 2006). To date, no domestication genes have been identified in the date palm. If different functional mutations can be identified in the same domestication gene for the North Africa and Gulf populations, then this would increase the likelihood that there were independent domestication events (Zohary 1999; Kovach *et al.* 2007). Efforts to identify domestication genes or to fully sequence multiple genomes across the date palm growing region (http://puruggananlab.bio.nyu.edu/index.php?action=100Dates) will be critical to our understanding of date palm domestication origins.

Our collection is biased toward the Arabian Gulf region, and ongoing collections are improving sample coverage from the western and southern edges of date palm cultivation. We do not exclude the possibility that analysis of additional cultivars could reveal further population structure. It is possible that evidence for a third center of early cultivation, especially between eastern and western North Africa, could emerge.

Low levels of breeding between Western and Eastern date palm cultivars are an interesting finding given the political history of the region. That is, despite large amounts of trade and movement across the region, especially during the Roman and Islamic empires, the two date palm groups have remained genetically distinct. We observe regions of admixture at the interface between North Africa and the Arabian Gulf, and this is expected given a model of two ancient centers of cultivation with enough time to become genetically distinct.

Linkage Group 12, harboring the putative sex determination region (Mathew *et al.* 2014), showed significantly higher numbers of alleles with frequencies that diverged between Western and Eastern cultivars. The date palm cultivars analyzed here were all female and the divergence observed is therefore between the X alleles. The nature of X chromosome replication, including lower effective population size and more time spent in the slower mutating female, suggests that diversity of X should be low within a population but F_st_, or fixation of those SNPs, will be high between populations (Schaffner 2004). Our findings to date have indeed suggested lower diversity (Al-Dous *et al.* 2011) and higher F_st_ (in this report) between the date palm subpopulations in scaffolds linked to the X chromosome. The differences in variability we observed in the putative date palm sex chromosomes could be associated with the differences in divergence of sex chromosomes compared to autosomes and would make the date palm a candidate for the “faster-X hypothesis.” This hypothesis explores the idea that sex chromosomes diverge at a different rate compared to autosomes (Charlesworth *et al.* 1987). It is proposed that mutations are fixed or eliminated at higher rates in X-linked loci, as selection acts more directly on X-linked genes in the hemizygous (XY) sex. The conditions under which this phenomenon occurs have been explored in different models, including *Drosophila*, chimpanzee, and humans (Betancourt *et al.* 2002). The debate on the theory remains open and the nature of date palm resources including a genome sequence, genetic map, and, now, genotyped cultivars for this XY-harboring plant species would provide a good model for further investigation of the theory.

Based on our observations that the date palm putative X chromosome has more rapidly fixed differences between the subpopulations of date palm cultivars, we propose that X-linked markers, combined with Y-linked markers, may be of interest for finer-scale geographical studies of date palm (Schaffner 2004; Cherif *et al.* 2013). Moreover, to our knowledge, this is the first report to use date fruit as a source of DNA on a large scale. The validated genotype results with respect to the genotypes collected from leaves should simplify the process of future date sample collections from the commercially important females.

Understanding the genetic structure among date palm cultivars will assist in selecting cultivars that span the major subpopulations for functional studies. Studies, such as those investigating date fruit nutritional values, have not typically taken into account the importance of regional variation in the fruit. Our findings here suggest that future studies should sample, at minimum, from the two major regions to better cover the natural date palm fruit differences, including fruit length, color, sugar content, and others. Work presented here will form an important foundation for genetic conservation and further functional analysis of the date palm genome.
